# Supplementary material for: Magnesium Plus Hydrogen Fertilization Enhances Mg Uptake, Growth Performance and Monoterpenoid Indole Alkaloid Biosynthesis in Catharanthus roseus
Source: Plants (Basel). 2025 Oct 31;14(21):3336. doi: 10.3390/plants14213336 (PMC12609934; doi:10.3390/plants14213336)
Supplement: Supplementary file 1 [file plants-14-03336-s001.zip › plants-3890275-supplementary figures and tables.pdf]

SUPPLEMENTAL INFORMATION

Supplemental Figures

Figure S1. *Catharanthus roseus* plants under four fertilizers treatment.

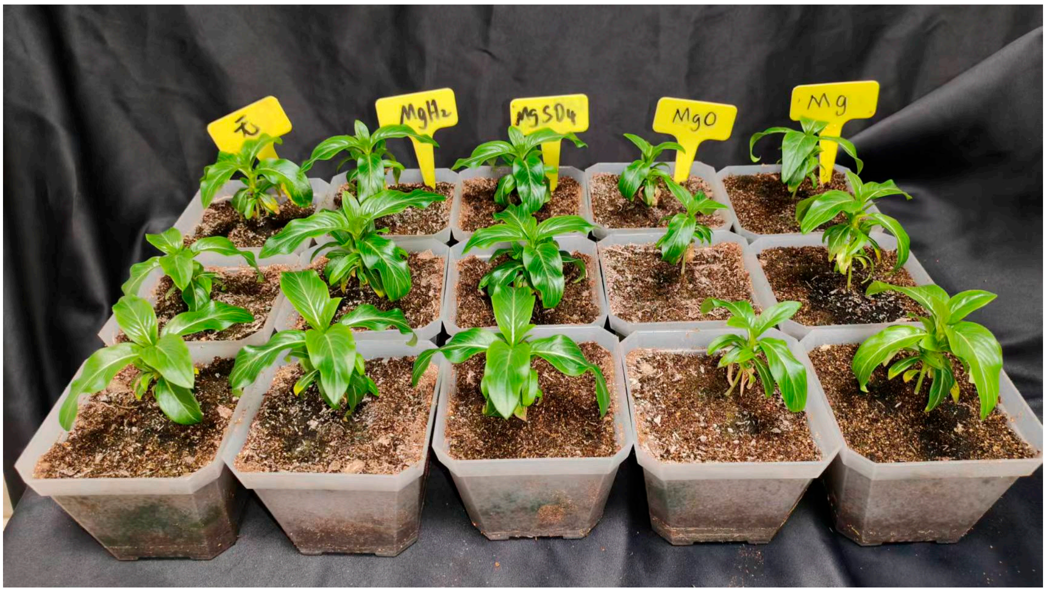

Figure S2. The calibration curves, linear regression equation and correlation coefficient (R^2) of catharanthine, vindoline and ajmalicine.

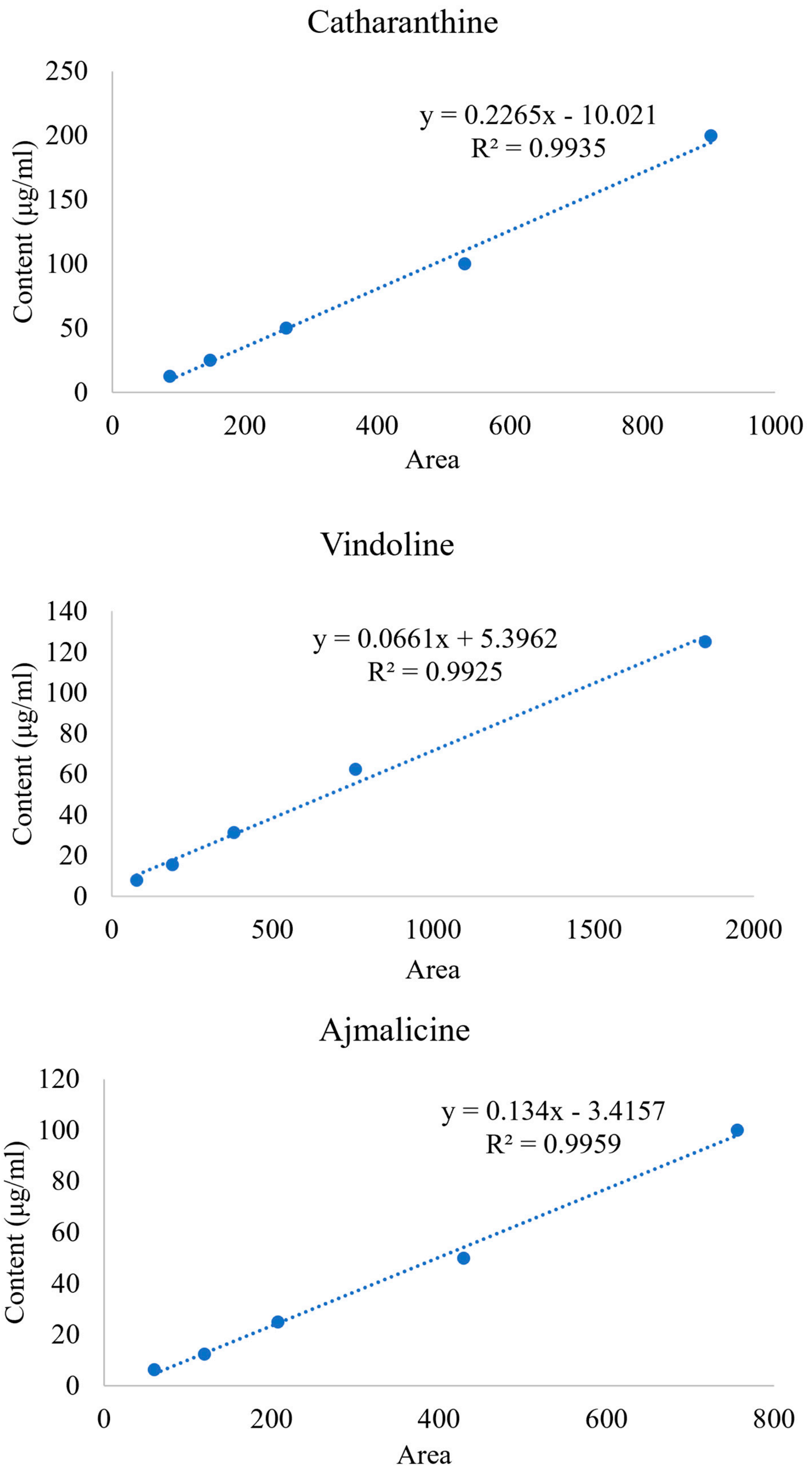

Supplemental Tables

Table S1. List of primers used in this work.

Primers	Purpose	Primer Sequence (5'-3')
N2227-F	qRT-PCR	GGTTGCTCTTCATTACGGATTT
N2227-R	qRT-PCR	TGCAGCATAGTAATGGTTTTGC
CrEIN3-FP	qRT-PCR	CTGTCCACCTTTGTCCTCAT
CrEIN3-RP	qRT-PCR	ATGTGGTTGTGGTTGACCTG
CrWRKY1-F	qRT-PCR	GTACTTGGTCCCGACGATATTC
CrWRKY1-R	qRT-PCR	CGAAACATTCCTTCGTTTGTAAG
CrMYC2-F	qRT-PCR	AGTGGTGAAGGAGGCAGAGA
CrMYC2-R	qRT-PCR	ATGGCTCTTCCCTTCCATTT
ORCA3-F	qRT-PCR	CGGGATCCGAAATACAGAAA
ORCA3-R	qRT-PCR	GCCCTTATACCGGTTCCAAT
CrERF5-F	qRT-PCR	CTCCATGGTTAAGGGGAGAGAT
CrERF5-R	qRT-PCR	ATGTACATTGGCCAAACACGGC
TDC-F	qRT-PCR	ATCCGATCAAACCCATACCA
TDC-R	qRT-PCR	CGTCATCCTCGACCATTTTT
STR-F	qRT-PCR	ACCATTGTGTGGGAGGACAT
STR-R	qRT-PCR	ATTTGAATGGCACTCCTTGC
SGD-F	qRT-PCR	GGAGGCTTCTTGAGTGATCG
SGD-R	qRT-PCR	GCAAATTCACCAGTGGCATA
GS-qF	qRT-PCR	GGTGGTAAGGCTACTGTTAT
GS-qR	qRT-PCR	GTGGTGTCCACAACACCATC
GO-qF	qRT-PCR	gtctggaacaccacttagat
GO-qF	qRT-PCR	GATGTCTGCATTAGTGATTCTG
Redox1-QF	qRT-PCR	GAAGTGACGGAAGTGGGGAACAAA
Redox1-QR	qRT-PCR	TCGCATTCGCCACATGAGTCAA
SAT-F	qRT-PCR	GGATGGGGAAAGCCTGTTTCTGTT
SAT-R	qRT-PCR	CTTCAGCCATGCTGATCCATGCTT
CS-QF	qRT-PCR	CTTCAGATGAGACTATTTGG
CS-QR	qRT-PCR	CTAGCGGAAACTTGTGATG
TS-F	qRT-PCR	TGCTCCTGGTGGAAATGATAACCC
TS-R	qRT-PCR	AATCAGCAACCTCGAGCAACCA
PAS-QF	qRT-PCR	TGGCTCAATTCCTGAAGCTT
PAS-QR	qRT-PCR	TTGGGTGATTTGAGGAATC
DPAS-qF	qRT-PCR	AACATCCCATTAAGGCTTACG
DPAS-qR	qRT-PCR	TTGAGGCAAGATCAGTGTG
PRX1-QF	qRT-PCR	ATTCAAGGTGCCCTGACTTG
PRX1-QR	qRT-PCR	TGACGGAGAGTCAAGTTAGG
T16H-qF	qRT-PCR	GCCCAAAACAGCCAATATTCAAACC
T16H-qR	qRT-PCR	ATGTGATGAGTATGGCCACCGC
16OMT-qF	qRT-PCR	AATGGGCATTTCTCTTTAAGGA
16OMT-qR	qRT-PCR	CCGTAATACAAATTGGGTACAA
T3R-F	qRT-PCR	CCCATGTGATGAGCAAAGCG

T3R-R	qRT-PCR	AACAATGGAGCAGGAGGGTG
NMT-QF	qRT-PCR	TTCGTGAGATGGTTCGGGTG
NMT-QR	qRT-PCR	CGGCGCCGTCACATATTTTT
D4H-QF	qRT-PCR	ATAGTTAATCATGGGATTCCACAAGATGTT
D4H-QR	qRT-PCR	GTTCATGAAACTTACGAACTCCATCTAC
DAT-QF	qRT-PCR	CTTCTTCTCATCACGTACCAACTC
DAT-QR	qRT-PCR	ATACCAAACTCAACGGCCTTAG

Table S2. List of qRT-PCR cycling conditions

Steps	Cycles	Temperature/℃	Time	Content
Initial denaturation	1	95	2min	Initial denaturation
PCR reaction	40	95	10s	Denaturation
		60	30s	Annealing
		72	30s	Extension
